# Insect Odorscapes: From Plant Volatiles to Natural Olfactory Scenes

**DOI:** 10.3389/fphys.2019.00972

**Published:** 2019-08-02

**Authors:** Lucie Conchou, Philippe Lucas, Camille Meslin, Magali Proffit, Michael Staudt, Michel Renou

**Affiliations:** ^1^INRA, Sorbonne Université, INRA, CNRS, UPEC, IRD, University P7, Institute of Ecology and Environmental Sciences of Paris, Paris, France; ^2^CEFE, CNRS, EPHE, IRD, Université de Montpellier, Université Paul-Valéry Montpellier, Montpellier, France

**Keywords:** insect olfaction, plant volatiles, odorscape, volatilome, olfactome, plant-insect interaction, landscape, sensory ecology

## Abstract

Olfaction is an essential sensory modality for insects and their olfactory environment is mostly made up of plant-emitted volatiles. The terrestrial vegetation produces an amazing diversity of volatile compounds, which are then transported, mixed, and degraded in the atmosphere. Each insect species expresses a set of olfactory receptors that bind part of the volatile compounds present in its habitat. Insect odorscapes are thus defined as species-specific olfactory spaces, dependent on the local habitat, and dynamic in time. Manipulations of pest-insect odorscapes are a promising approach to answer the strong demand for pesticide-free plant-protection strategies. Moreover, understanding their olfactory environment becomes a major concern in the context of global change and environmental stresses to insect populations. A considerable amount of information is available on the identity of volatiles mediating biotic interactions that involve insects. However, in the large body of research devoted to understanding how insects use olfaction to locate resources, an integrative vision of the olfactory environment has rarely been reached. This article aims to better apprehend the nature of the insect odorscape and its importance to insect behavioral ecology by reviewing the literature specific to different disciplines from plant ecophysiology to insect neuroethology. First, we discuss the determinants of odorscape composition, from the production of volatiles by plants (section “Plant Metabolism and Volatile Emissions”) to their filtering during detection by the olfactory system of insects (section “Insect Olfaction: How Volatile Plant Compounds Are Encoded and Integrated by the Olfactory System”). We then summarize the physical and chemical processes by which volatile chemicals distribute in space (section “Transportation of Volatile Plant Compounds and Spatial Aspects of the Odorscape”) and time (section “Temporal Aspects: The Dynamics of the Odorscape”) in the atmosphere. The following sections consider the ecological importance of background odors in odorscapes and how insects adapt to their olfactory environment. Habitat provides an odor background and a sensory context that modulate the responses of insects to pheromones and other olfactory signals (section “Ecological Importance of Odorscapes”). In addition, insects do not respond inflexibly to single elements in their odorscape but integrate several components of their environment (section “Plasticity and Adaptation to Complex and Variable Odorscapes”). We finally discuss existing methods of odorscape manipulation for sustainable pest insect control and potential future developments in the context of agroecology (section “Odorscapes in Plant Protection and Agroecology”).

## Introduction

Olfaction is a very important sensory modality for insects and volatile organic compounds (VOCs) serve as chemical cues to recognize and locate vital resources such as food, mate, or enemies. Insects live in a very complex chemical world from which they must extract this relevant information. Considerable progresses in sensitivity and selectivity of analytical methods have allowed to identify minute amounts of the semiochemicals that mediate a wide variety of insect-insect or insect-plant interactions. Yet, we do not possess a global envision of the chemical environment insects live in.

It has long been acknowledged that animals experience their own species-specific sensory world. As early as 1934, Jakob von Uexküll defined the Umwelt (von Uexküll, 1934) as the subjectively perceived surroundings about which information is available to an organism through its senses. In neuroethological terms, the Umwelt corresponds to the range of stimuli the insect’s receptor set can detect and translate into a neural code which is further interpreted in the brain to finally trigger the appropriate physiological or behavioral response. This notion of a sensory world proper to a species appears particularly appropriate for olfaction since individual olfactory receptors (ORs) detect only a small fraction of existing volatile chemicals and considerable variation has been documented in the number and tuning of ORs expressed by different insect species. Accordingly, the odorscape can be defined as the ensemble of the VOCs that constitute a sensory space proper to a particular insect species.

The VOCs constituting the insect odorscape may serve different ecological functions independently of their chemical nature. Volatile signals are chemicals that are produced by a living organism with the function of exchanging information with other living organisms. For instance, a plant attracts pollinators by advertising for the presence of a reward, e.g., nectar, when its flowers are receptive; a molested aphid emits an alarm pheromone inducing escape in congeners; or a female moth at sexual maturity signals herself to conspecific males by releasing a volatile sex pheromone. On the other hand, cues carry information about the availability of a resource to the receiver, although they are not emitted for a communication purpose and can be released by a lifeless source. Receivers must extract signals and cues from a background composed of many other VOCs, which might alter their perception. For instance, the ability of *Manduca sexta* moths to locate their host plant is significantly decreased in a background of benzaldehyde or geraniol compared to host plant odor alone (Riffell et al., 2014).

Plant and insect species live in close intimacy with each other. The standing biodiversity of both taxa is to a large extent the result of their ancient co-evolution (Labandeira, 2007; Labandeira et al., 2007). Insects depend on plants as food sources either directly for phytophagous and pollinator species or indirectly for parasitoids and predators. This explains the importance of volatile compounds of plant origin (volatile plant compounds or VPCs) for insect ecology. Almost all kinds of plant tissues (leaves, flowers, fruits, roots, etc.) and types of vegetation (trees, grasses, shrubs, etc.) release VPCs albeit with different profiles and in different amounts. Moreover, plants make up most of the biomass of most terrestrial ecosystems, making them the major source of biogenic volatiles and therefore of insect odorscapes. Overall, the terrestrial vegetation produces and releases an amazing variety of volatiles including isoprenoids, benzenoids, oxygenated low-molecular weight VOCs, sulfur-containing compounds, fatty acid-derived volatiles, etc. These VPCs can be emitted either constitutively or in response to a variety of abiotic and biotic stimuli or stresses. They are involved in a wide variety of ecological functions. They can for instance protect plants against abiotic stress and mediate plant-plant and various plant-animal interactions (Unsicker et al., 2009; Loreto and Schnitzler, 2010). Moreover, due to their high abundance and reactivity, VPCs drive air chemical processes that affect air quality and climate at regional and global scales, affecting plant growth in return (Penuelas and Staudt, 2010).

Finally, it is expected that the atmospheric content in VPCs varies locally according to the composition of the plant communities specifically associated to natural habitats or agricultural landscapes, resulting in the perception by insects of distinct “odorscapes.” The concept of a sensory-scape has been first used for physical sensory modalities. The term soundscape was noted by Michael Southworth in his 1969 article titled “The Sonic Environment of Cities” (Southworth, 1969) and developed in more detail 8 years later by Canadian composer and naturalist R. Murray Schafer in his seminal work, “Tuning of the World” (Schafer, 1977). Some years after Bernie Krause contributed by his recordings to the emergence of soundscape ecology (Pijanowski et al., 2011). Indeed the term “scape” adds a notion of spatiality, or spatial determination to a sensory word. Concerning olfaction, the term odor-landscapes has been used to describe the spatiotemporal distribution of chemical concentrations resulting from their propagation in fluid media (Atema, 1996; Moore and Crimaldi, 2004). The concept of landscape also involves a notion of movement by the receptor organism and the stimuli detected by an insect are changing as it moves in its milieu. Due to variations in the emission rates, the physical transportation, and interception on surfaces and chemical degradation, the distribution of VPCs in the insect environment is heterogeneous in space and varies in time, which in addition to chemical complexity makes describing the fine structure of odorscapes particularly challenging. This review aims to better apprehend the olfactory environment of the insect in its chemical (sections “Plant Metabolism and Volatile Emissions” and “Insect Olfaction: How Volatile Plant Compounds Are Encoded and Integrated by the Olfactory System”), spatial (section “Transportation of Volatile Plant Compounds and Spatial Aspects of the Odorscape”), temporal (section “Temporal Aspects: The Dynamics of the Odorscape”), ecological, and cognitive (sections “Ecological Importance of Odorscapes” and “Plasticity and Adaptation to Complex and Variable Odorscapes”) dimensions. We also discuss how a better knowledge of insect odorscapes may benefit sustainable crop protection (section “Odorscapes in Plant Protection and Agroecology”).

## Plant Metabolism and Volatile Emissions

Plants produce a bewildering variety of VOCs comprising a great diversity of chemical structures. Volatility is measured by vapor pressure and is limited by the molecular weight (around 300 g) and also depends on the polarity of the chemical structure. A large majority of VPCs stem from four different metabolic pathways: the mevalonic acid (MVA) and methylerythritol phosphate (MEP) pathways for isoprenoids, the lipoxygenase (LOX) pathway for fatty acid derivatives, the shikimic acid pathway for benzenoids and phenylpropanoids, and the amino acid derivatives pathway (Baldwin, 2010). In addition, diverse metabolic paths produce various alkenes and low-molecular weight oxygenated compounds like ethylene, acetaldehyde, acetone, or methanol (Jardine et al., 2017) that may play a role in insect-plant interactions. Some VPCs are ubiquitously emitted from a wide range of plant species while others are released only from specific plant taxa. Hence, the composition of volatile emissions typically differs between plant species. Secondary metabolites in general have been extensively used in plant classification (chemotaxonomy) and modern algorithms for data analyses confirm the close relationship between the volatile metabolome and plant taxonomy (Vivaldo et al., 2017).

However, VPC emissions are also highly variable within a plant species. Since all VPC metabolic pathways do not respond in the same way or with the same intensity to biotic and abiotic factors, the amounts and the composition of volatiles released from a given plant species can strongly vary with environmental conditions, including plant-plant interactions, above or below ground (Delory et al., 2016). Some plant volatile emissions are stimulated by the attack of an herbivorous insect and serve as chemical weapons to protect plants against these attacks (Unsicker et al., 2009; Dicke, 2016; Rowen and Kaplan, 2016) either directly, or by attracting their natural enemies. For instance, species of the *Allium* genus such as the leek (*Allium porrum*) produce alk(en)yl-cysteine sulfoxides that are precursors of volatile thiosulfinates and disulfides (Dugravot et al., 2005). The production of these sulfur-containing VPCs increases sharply after attack by the leek moth, *Acrolepiopsis assectella*, a specialist feeder. Attacked leek plants are not avoided by the moth but females of *Diadromus pulchellus* an endoparasitoid wasp of young moth chrysalids, are more strongly attracted to damaged leek. In addition, the frass of *A. assectella* larvae contains dimethyl disulfide, dipropyl disulfide, and methyl-propyl disulfide that attract the wasps (Dugravot and Thibout, 2006). In poplars (*Populus nigra*) attacked by *Lymantria dispar* caterpillars, a clear increase of nitrogenous and aromatic compounds has been observed in the volatile emissions (McCormick et al., 2014). Even plant-associated microorganisms such as epiphytic bacteria on flowers and leaves can significantly affect the VPC composition released by a plant organ (Helletsburger et al., 2017).

Within a plant, the composition of the emissions can largely differ among organs and may vary with the circadian rhythm, the plant’s age and phenological state (maturity, senescence, etc.) (Hare, 2010). For example, a recent field study on VOC emissions from maritime pine revealed that pinene emissions from branches have a distinctly different enantiomeric signature (optical isomers) than pinene emissions from the stems of the same trees (Staudt et al., 2019). Such a minute diversification in VPC production may have important ecological implications, since many insects such as bark beetles possess stereo-selective ORs that distinguish between optical VPC isomers (Andersson, 2012).

## Insect Olfaction: How Volatile Plant Compounds are Encoded and Integrated by the Olfactory System

**Figure 1:**
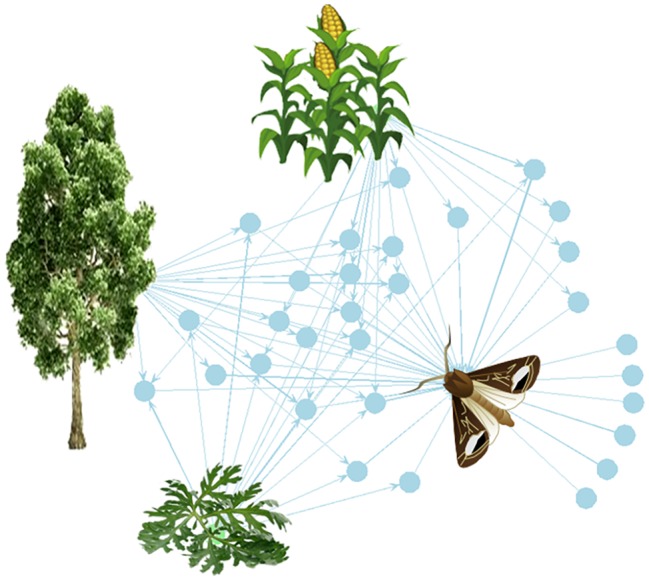
The plant-VPC-insect network. The complex odorscape of a moth (*Agrotis ipsilon*) shown as a communication network between plants, VPCs, and insect spaces. The network graph is based on the chemical analyses of plant VPCs and recordings of EAG responses from the literature (Greiner et al., 2002; Jerkovic et al., 2003; Degen et al., 2004; McCormick et al., 2014). It has been drawn using the R package “network” (R Core Team, 2013). The plant species release VPCs (blue spots) that are detected by the olfactory system of the moth. The information contained in VPCs circulates from the plants to the insect (blue arrows). Each plant emits a variety of VPCs and one VPC can be produced by different plant species. For simplification, the VPCs not detected by the moth have been omitted. The olfactory system of the moth detects VPCs released by its host plant, maize, as well as by companion plants, such as a weed (*Artemisia vulgaris*) and trees surrounding the fields (*Populus nigra*). Most of the VPCs are shared between two or three plants. This simplified network does not take into account the intensity of the emissions, which can largely differ among VPCs released by a same plant, and moreover varies according to the biomass of individual plants and of the whole plant communities.

A VPC only becomes an odor if it gets detected by a biological sensor, here the olfactory system of an insect. Thus, to the volatilome of plants corresponds the olfactome of insects, the spectrum of all the volatile compounds that are detected by a species (Figure 1). In this section, we briefly examine how volatile compounds are detected by the insect olfactory system. Insect olfaction is a complex sensory process that runs from the specific detection by binding onto ORs expressed in olfactory receptor neurons (ORNs) to neural code, blend perception (integration in brain), and behavior.

The stimulus quality is first encoded in the pool of odorant-binding proteins (OBPs) and ORs expressed in the olfactory organs of a given insect species. While OBPs are generally thought to play an important role in the solubilization and transport of the odorants (Pelosi et al., 2017), here we will only focus on the ORs, as they are the molecular actors that trigger the olfactory signaling cascade. ORs are hosted by ORNs enclosed inside the olfactory sensilla on the antennae and palps of insects. Typically, ORs have a seven-transmembrane topology and form heterodimers with a co-receptor named Orco (Butterwick et al., 2018). While Orco is fully conserved across species, sharing up to 94% sequence identity with orthologs from different species (Butterwick et al., 2018; Soffan et al., 2018), the sequence identity of the other ORs can vary greatly within and between species (Hansson and Stensmyr, 2011). Additionally, the size of the OR repertoire, i.e., the content of OR sequences in a genome, differs from one species to another, reflecting their ecology and lifestyle as well as the evolutionary history of the species. Extreme examples of OR repertoire sizes can be found among parasitic and social insects. The human body louse (*Pediculus humanus*), an obligate parasite with a very specific ecological niche with a relatively constant environment, has only 10 ORs (Kirkness et al., 2010). On the contrary, some ants, that have a complex social organization in which olfaction plays an important role and that are exposed to various environment, express up to 350 ORs (Zhou et al., 2012). However, in addition to the size of the OR repertoire, the chemical tuning of each individual OR is fundamental to evaluate the olfactory capacity of an insect species. Indeed, the capacity of each OR to detect volatiles varies in terms of specificity and sensitivity. Specialist ORs have a very narrow binding spectrum. It is usually the case for the ORs that bind pheromone compounds. Other ORs are more broadly tuned. In addition, the spectrum width increases with odorant concentration, the ORs being more narrowly tuned at low concentrations (de Fouchier et al., 2017). Combinatorial coding, where each odorant activates a different set of ORs, allows the discrimination of a greater number of odorants than the number of OR types, increasing the olfactory capacity of an individual insect. Finally, different subsets of ORs are expressed depending on sex or life stage, in order to accommodate the ecological needs of individuals (Poivet et al., 2013). The size of the repertoire, the diversity, the tuning, and the timing of expression of ORs make the description of the olfactome a complex task. Owing to the generalization of DNA and protein sequencing methods, an increasing amount of OR sequences is now available. The deorphanization of receptors is the limiting step, requiring heavy and sophisticated techniques. The development of high-throughput methods should play a crucial role in the upcoming years in this essential step to get a sense of the true olfactome of each species. Furthermore, the notion of OR repertoire should be treated in all its complexity. Indeed, while the sequence information is important, it is not sufficient. Major shifts in the olfactory system can be achieved through a change in the expression of ORs, or even a change in the neuronal projection to the brain (Dekker et al., 2006; Kopp et al., 2008; Tait et al., 2016).

The firing response of insect ORNs is proportional to the aerial concentration of odorants and has a much wider dynamic range than that of their vertebrate counterparts (Rospars et al., 2014). This intensity coding informs insects on the absolute levels of odorants in the atmosphere and allows detection of changes in aerial concentrations. However, because of turbulences in natural conditions, the odor plume does not form a continuous gradient pointing to its source (Celani et al., 2014), making odor navigation at large distances a challenging task. This point will be discussed more in section “The Odorscape as a Source of Spatial Information: Habitats, Trails, and Landmarks”.

All ecologically relevant sources release odor blends rather than individual odorants. Perception of blends of VPCs plays a pivotal role in the recognition of the host plant and avoidance of non-host plants (Bruce and Pickett, 2011; Cha et al., 2011). Blend recognition involves the sensory integration of the information carried by ORNs at different levels in the insect brain, the antennal lobe and the protocerebron (Silbering and Galizia, 2007; Galizia and Rössler, 2010; Galizia, 2014). Not only does the nature of each component matter, but also its proportion in the blend. Detailed investigations on blend perception, for instance in bees, have shown that in some cases the insect still perceives the individual components (elemental processing), but most often a distinct entity is perceived (configural processing) (Deisig et al., 2006). In addition, interactions between the components of a blend can lead to a reduced perception of the blend, compared to that of its individual components, a process termed “mixture suppression” (Ache et al., 1988). Thus, olfaction is a highly integrative sensory process which makes the prediction of insect responses in complex odorscapes difficult. Furthermore, multi-modal integration, for instance between vision and olfaction, can increase responses to odorants (Strube-Bloss and Rossler, 2018).

Eventually, the odor perception may trigger a conspicuous change in insect behavior. Male moths, for instance, are sexually aroused and attracted by the female-emitted pheromone. They take flight and navigate the pheromone plume upwind toward the source performing chemically triggered anemotaxy (Cardé and Willis, 2008). This behavior is innate. However, because of the integration in the insect brain of the complex olfactory input from a pool of ORNs, the detection of an odorant does not preclude of the type of behavior that follows. The males of several noctuid moth species also possess ORNs specifically tuned to some of the components of the pheromones produced by females of sympatric species. Activation of these ORNs inhibits their attraction to their own pheromone (Berg and Mustaparta, 1995; Berg et al., 2014). For plant volatiles, it has long been acknowledged that phytophagous insect species sharing large parts of their olfactome nevertheless show different preferences in their behavioral responses to host plant volatiles (Bruce et al., 2005). In other words, the full knowledge of the olfactome will not suffice to predict the behavior.

Insects can also be innately repelled by specific odorants. Geosmin, an earthly smelling substance of bacterial origin, deters oviposition by *Drosophila melanogaster* females, preventing them from laying their eggs on fruits colonized by harmful molds (Stensmyr et al., 2012). Based on such attraction or avoidance behaviors, the concept of valence is commonly applied to insects. Valence value is considered as positive when the insect is attracted, negative when it is repelled. This does not postulate a hedonic value, which could be questionable in arthropods. It seems essential to determine whether odor valence conserves the same value among species, is stable during an individual life, and is treated in specific neuronal circuits according to its value. Since it has long been acknowledged that the same odorant may attract some, while repelling other insect species, it is easy to confirm that valence pertains to the species. Isothyocyanates for instance, repel generalist herbivores but attract *Brassicaceae* specialists (Hopkins et al., 2009). Several bark beetle species avoid hexanol isomers and monoterpenes associated with deciduous non-host trees, while the same molecules are attractive to insects feeding on deciduous trees and their parasitoids (Zhang et al., 1999; Byers et al., 2004). Carbon dioxide (CO_2_) is a salient odorant for many insects (Guerenstein and Hildebrand, 2008). CO_2_ attracts hematophagous insects seeking for a host (Stange, 1996). The tobacco hawk moth *M. sexta* is attracted to elevated CO_2_ levels emitted from fresh opening flowers of *Datura wrightii* (Solanaceae) that likely contain large amounts of nectar (Thom et al., 2004). By contrast, CO_2_ elicits innate avoidance in *Drosophila* (Suh et al., 2004) but this behavior is context dependent, testifying that valence may change during the life of an individual. Indeed, *Drosophila* prefers feeding on rotting fruits that emit CO_2_ as a by-product of fermentation by microorganisms and yeasts. Two compounds, 2,3-butanedione and 1-hexanol, present in *Drosophila* food sources, but more abundant during fruit ripening, strongly inhibit the response of CO_2_-sensitive ORNs by direct interaction with the CO_2_ receptor, suppressing the avoidance of CO_2_ by flies (Turner and Ray, 2009). The valence of an odorant often changes with its concentration. *D. melanogaster* for instance shows an innate and robust attraction to vinegar but higher concentrations of vinegar are less attractive (Semmelhack and Wang, 2009). Experimental evidence establishing the localization of valence treatment in the insect olfactory system is scarce. For instance, no valence-specific activation of ORNs was found in *Drosophila* flies, but the categorization of odors as pleasant or unpleasant seems to be established at the antennal lobe level (Knaden et al., 2012) and might be maintained from the antennal lobe to the lateral horn (Min et al., 2013).

To conclude this brief overview of the insect olfactory system, the behavioral activity of a given odorant is not only odorant dependent but also receptor, species and context dependent. This level of complexity calls for integrative approaches from gene to behavior in order to understand what insects smell.

## Transportation of Volatile Plant Compounds and Spatial Aspects of the Odorscape

Because insect behavior depends on how volatile compounds are distributed in space and time, insect chemical ecology has very early paid attention to the processes that determine odorant fate in the atmosphere (Riffell et al., 2008). The dispersion of the odorant molecules in the atmosphere depends on the characteristics of the sources, the importance of the compartments where they can be sequestrated (sinks), and the physical laws describing fluid movements. Biological sources of volatile compounds are considerably variable in their emission capacity. As the cuticle area above the pheromone gland of a female moth is in the range of tens of square micrometers, a single female may be approximated as a point source. On the contrary, the billions of yellow flowers from a blooming field of rape constitute a huge source of floral volatiles covering hundreds of square meters. VPC exchanges have been analyzed only in a few agricultural ecosystems. In a maize field, fluxes at ecosystem scale show large differences between families of VPCs, 8 ± 5 μg m^−2^ h^−1^ for isoprene and 4 ± 6 μg m^−2^ h^−1^ for monoterpenes but 231 ± 19 μg m^−2^ h^−1^ for methanol (Bachy et al., 2016). Once released in the air, the pheromone and the crop plant volatile molecules are carried by air flows according to identical physical processes but generate plumes with different shapes and dimensions. The pheromone forms a meandering plume, roughly cone shaped, with its main dimension in the wind axis. Such a narrow plume builds a chemical trail that insects can follow upwind. The physical structure of the plume has been analyzed and modeled, revealing a statistical distribution of pheromone molecules into intermittent filaments (Celani et al., 2014). The emissions of an individual plant, or plant organ, behave probably much like a pheromone, enabling the insect to fly back up the source. By comparison, the dispersion of VPCs at field scale has not been so finely investigated but one can expect that it builds up local odorant ambiances that resolve into broad downwind odor cones. Thus, odorscapes can be seen in space as trails and scenes, similar to the paths and sceneries of a physical landscape, although less stable. In such odorscapes, insects can stay on a spot and be durably exposed to local high concentrations, move to a more suitable habitat, or navigate an odor plume.

Most of the physics beyond the distribution of odors is known from environmental fluid mechanics. Volatile organic compounds spread away from their source through molecular diffusion and through transportation by wind and other air flows. Diffusion is the net movement of molecules from a region of high concentration to a region of low concentration as a result of their random motion. It is slow and acts significantly only at very small distances (below 1 m). Its contribution to the distribution of VPCs at field scale is therefore much smaller than that of air transport. By contrast, diffusion might be the major driver of VPC movements in the soil.

Wind is of course the strongest factor of horizontal dispersion of odors in natural landscapes but in fact, three types of air movement co-occur: advection, convection, and turbulence, resulting in transport and dilution of VPCs not only horizontally, but also vertically. Advection is the bulk transport of a substance by a flow: the wind carries away the VPCs horizontally, quickly, and on long distances. Convection is the vertical transport by thermals, finite parcels of fluid consisting in the same fluid as its surroundings but at a different temperature. Differences in air temperature or moisture can lead the atmosphere to stratify in layers of different densities, which limits vertical transport of VPCs. Differences in air density may be stable, which for example explains smog episodes over cities. Finally, turbulences may arise from three main mechanisms. When the wind encounters physical obstacles like bushes or trees, local vortices or turbulences are generated. Shear turbulence is created by a flow scrubbing against a rough surface like the ground. Surface roughness depends on the vegetation cover and hence shear turbulence is different over bare soils, grasslands, crop fields, and forests. Convective turbulence is created by rising/sinking thermals. All turbulent flows cause stirring of odor pockets with the formation of eddies of different diameters and speeds. Eddies are then transported horizontally by advection. Turbulences cause intermittency in odorant signals, with pockets of odorized air separated by clean air, and favor mixing between ambient air and air carrying the signal. Introduction of ambient air into the plume dilutes odorants and mixes odorants from different sources or with airborne oxidants. For instance, Riffell et al. (2014) showed that the ratio of volatiles in the plume emanating from flowers of *Datura wrightii* changed with distance, as the background volatiles from neighboring vegetation, including creosote bush plants, became intermixed with *D. wrightii* volatiles. The average VPC concentration decreases as the square of the distance from the source but local conditions can alter this rule. At landscape scale, local topography can favor the build-up of high levels of VPCs through valley or basin effects.

Although an odor emanating from a discrete source is typically represented as a scent cone produced by a moving fluid entering a quiescent body of the same fluid, the actual shape of an odor is dictated by air movements and must be seen as a plume (Murlis et al., 1992). Turbulences and random changes in the wind direction will cause this plume to meander, resulting in a “chemical trail.” Insects such as moths can fly over several hundreds of meters navigating upwind through such pheromone plumes (Shorey, 1976; Cardé and Charlton, 1984; Elkinton et al., 1987). An insect flying through a diversified landscape will experience areas differently odorized, both with respect to the nature of the volatile compounds and their mixing rates. Furthermore, vertical stratifications in the distribution of VPCs have been observed in many ecosystems. For example, in a neotropical forest, sesquiterpenes were most abundant in the air near the ground, whereas monoterpenes prevailed at higher canopy levels (Jardine et al., 2011, 2015). Finally, while we have gained a better knowledge of odor distribution at the field scale, the micro-distribution of VPCs at the scale of an insect’s body size (millimeters to centimeters) remains to be investigated. One should expect a large heterogeneity in accordance with the diversity of microhabitats created by the plant cover.

## Temporal Aspects: The Dynamics of the Odorscape

**Figure 2:**
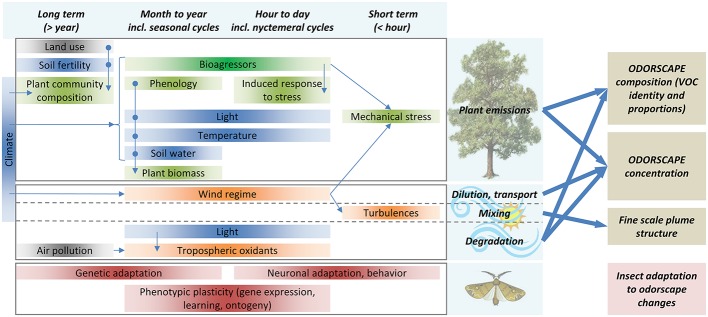
Odorscapes are highly variable at a diversity of time scales. The left part of this chart depicts the factors that determine volatile emissions by plants (top), atmospheric processes (middle), and the processes by which insects can adapt to changes in their olfactory environment (bottom), classified horizontally by the time scale at which they act or vary. Thin arrows depict indirect impacts, through influence on another factor. Box color: dark blue = climate and soil factors, green = plant physiology and ecology, orange = atmospheric physico-chemistry, brown = insect adaptation processes, gray = anthropic factors. Although this is not represented for readability issues, extremes of most climate and soil factors do cause stress responses in plants. VOC sources other than plants (especially anthropogenic VOC) are ignored. On the right side, thick arrows depict how plant emissions and atmospheric processes determine the characteristics of the odorscape.

Because of the variations in emissions, sinks, and atmospheric transport of VPCs, the odorscape composition changes considerably at seasonal, daily, hourly, and even minute to second time scales (Figure 2). A survey of VOCs in the atmosphere of rural and urban districts in Great Britain has shown that the maximum concentration of some VOCs may reach values 100 times higher than average, even above rural areas (Cape, 2003). Measures of fluxes over crop fields, forests, and other plant communities have all shown variations at all time scales listed above (Bachy et al., 2016; Schallhart et al., 2016).

### Temporal Variations in Emission

Emissions of VPCs can occur by sudden and short bursts. Plants respond to herbivory by rapidly modifying their emissions after attacks (Maffei et al., 2007). Wounding triggers bursts of so-called green leaf volatiles formed from the enzymatic breakdown of membrane lipids through the lipoxygenase pathway (LOX). The emission response is almost instantaneous and lasts only a few hours (see e.g., Staudt et al., 2010). Likewise, in plants that store VPCs in their tissues (e.g., essential oil in aromatic plants or oleoresin in conifers) or VPC precursors (e.g., glucosinolates in *Brassicaceae* or cyanogenic glucosides in *Rosaceae*), mechanic stress and injuries including herbivore attacks induce immediate emission bursts. However, the induction of many other stress-related VPCs is associated with gene activation and the resulting metabolic adjustments, which proceed over hours and days (Arimura et al., 2008).

Temperature positively drives both physical and physiological processes, leading to marked daily emission changes. In addition, the emissions of many VPCs are light dependent, because their biosynthesis is tightly linked to photosynthetic processes that deliver primary carbon substrates and biochemical energy. Foliar isoprene emissions, for example, cease at night. During the day, they can fluctuate rapidly, in response to changes in cloud cover and shading by the canopy (Singsaas and Sharkey, 1998). Light influence on stomatal conductance constrains emissions of polar water-soluble VPCs such as methanol, which can be further modified by diurnal changes in transpiration and water transport (Rissanen et al., 2018). On the other hand, emissions of apolar hydrophobic VPCs are independent of stomatal conductance, even though these VPCs diffuse principally through stomata (Niinemets et al., 2002). In addition to exogenous factors, an endogenous clock also controls dial emission variations. Photo-positive and less frequently photo-negative endogenous circadian rhythms have been reported for constitutive and stress-induced VPC emissions (Zeng et al., 2017).

Environmental factors exert various longer term effects on VPC emissions, independently of the aforementioned short-term modulations. Weather conditions and particularly the prevailing temperature regimes continuously up- and down-regulate the VPC-producing metabolism in plants (Fischbach et al., 2002; Staudt et al., 2003). Seasonal drought events can positively and negatively modulate VPC emissions, depending on the stress intensity (Staudt et al., 2008). Phenology is also a major driver since VPCs are mostly emitted from leaves and flowers that are absent or physiologically inactive during specific periods (Filella et al., 2013). The main periods of low emissions correspond to the cold season in temperate and subpolar climates, and to the dry season in arid subtropical climates. During these seasons, a large majority of insects, which depend directly or indirectly on plants as food sources, are inactive.

At decennial time scale, human activity and climatic change have profoundly modified natural odorscapes in the past and will continue to alter VPC emissions in the future at local, regional, and global scales (Lathière et al., 2010). Interestingly, these long-term changes in quantity and composition of VPC emissions can feedback on climate evolution since their reaction products in the atmosphere influence ozone concentrations and other parameters that will alter the balance between insolation absorbed by the Earth and the energy radiated back to space (e.g., Harper and Unger, 2018). Rising temperature and atmospheric CO_2_ concentration will directly affect VPC biosynthesis in addition to changing plant growth rates, phenology, and the length of the vegetation periods (Penuelas and Staudt, 2010; Staudt et al., 2017). Furthermore, the frequency and intensity of heat spells and drought stress will increase in some regions, potentially increasing the proportions of stress-induced VPCs in the atmosphere. Land use itself can deeply alter the odorscape, not only by changing profoundly the type and size of VPC sources (Hantson et al., 2017), but also by affecting the microclimate and the strength of VPC sinks.

### Atmospheric Degradation of Volatile Plant Compounds

**Figure 3:**
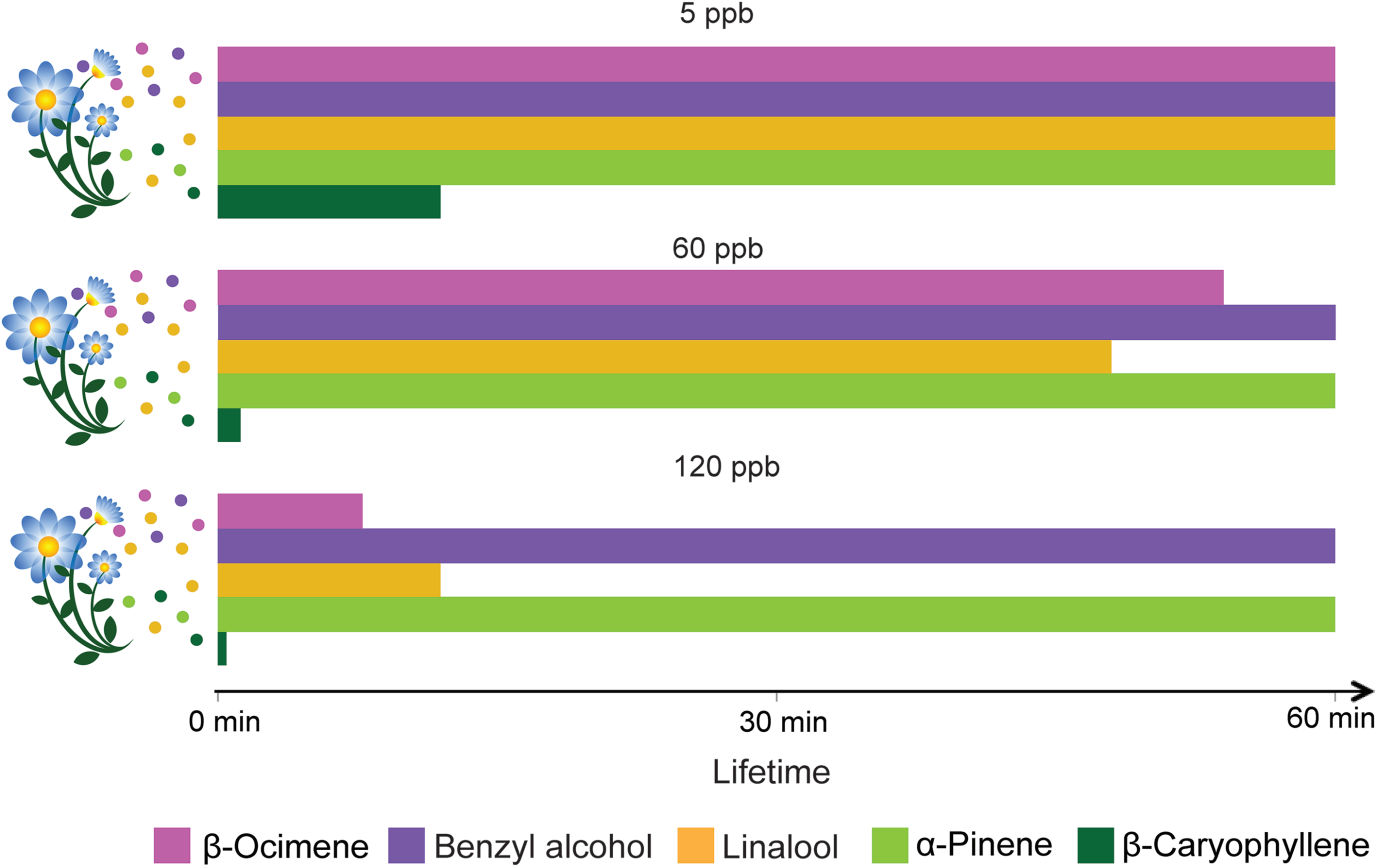
Effect of ozone concentration on odor plume composition over time. Lifetimes in the atmosphere, measured up to 60 min, of volatiles emitted commonly by flowers (β-ocimene, benzyl alcohol, and linalool) and by leaves (linalool, α-pinene and β-caryophyllene) are presented under the influences of the O_3_ levels indicated above the histograms. The degree of O_3_ reactivity is based on the structural properties of the VPCs (Atkinson and Arey, 2003).

Physical, chemical, and biological sinks limit the mixing ratios of VPCs in the air. Soils, for instance, can act as sinks for VPCs through mechanisms of dissolution and adsorption onto organic, mineral, and aqueous surfaces, and through degradation by aerobic and anaerobic microorganisms (Insam and Seewald, 2010). VPCs can also be deposited on or taken up by plants and eventually metabolized (e.g., Karl et al., 2010). However, the main VPC sink remains their atmospheric chemical degradation through reaction with atmospheric oxidants (Figure 3). The main oxidants are the hydroxyl radical (OH), ozone (O_3_), and the nitrate radical (NO_3_). O_3_ and the OH radical are secondary pollutants predominantly photochemically formed, essentially under sunlit conditions (Atkinson and Arey, 2003). In the troposphere, O_3_ is produced from the photolysis of NO_2_ to NO and a triplet O, the latter reacting with O_2_ to form O_3_. The OH radical – often referred to as the “detergent” of the troposphere – is formed from the photolysis of O_3_ to O_2_ and singlet O, which further reacts with a water molecule yielding two OH radicals. The nitrate radical is produced from the reaction of O_3_ with NO_2_ leading to O_2_ and NO_3_. The NO_3_ radical is considered an important oxidant only at night, because it photolyzes rapidly during the day. The reactivity of VPCs to these oxidants and their resulting atmospheric lifetimes are highly variable. For example, the O_3_ reactivity of benzyl alcohol, linalool, and β-caryophyllene differs by more than three orders of magnitude (Atkinson and Arey, 2003). The sesquiterpene β-caryophyllene is so reactive that under most conditions, it will last only a few minutes (Figure 3). The products of VPC reaction with atmospheric oxidants (mostly addition at double bonds) will first yield transitory unstable intermediates (radicals and ozonides) that rapidly react further to produce more stable oxygenated derivatives such as ketones, aldehydes, and organic nitrates. These secondarily formed oxygenated VPCs are generally less reactive than the primary ones (Atkinson and Arey, 2003). The number of hypothetical products formed from VPC-oxidant reactions increases exponentially with the number of carbon atoms present in the VPC molecules (Goldstein and Galbally, 2007). As a result, the initial bouquet of emitted VPCs becomes gradually mixed with a characteristic blend of its numerous degradation products during its aerial transport. The extent to which such secondary VPCs affect insect behaviors is not yet well understood, even though impairment of insect orientation has been reported. For instance, primary pollutants in diesel exhaust (Girling et al., 2013) can differentially degrade floral VPCs and affect the foraging efficiency of honeybees. Similarly, laboratory experiments with herbivorous insects, bumble bees, and parasitoids indicated that realistic O_3_ concentrations impair insect attraction to their host plants (Fuentes et al., 2013, 2016; Farré-Armengol et al., 2016). A modeling framework was used to simulate the modification of floral scent plumes by dispersion and chemical degradation and its impact on foraging pollinators (Fuentes et al., 2016). Even moderate levels of air pollutants (e.g., 60 ppb O_3_) can substantially degrade floral volatiles, increase the foraging time of insects, and reduce their ability to locate host plants. The study also highlights that plant-pollinator interactions could be sensitive to changes in floral scent composition, especially if insects are unable to adapt to the modified odorscape.

The atmospheric concentration of oxidants varies temporally and spatially. In particular, OH and NO_3_ radical concentrations show pronounced diurnal variations, though in opposite trends. In addition, the intensity of turbulent transport is usually different during day and night. At night, the tropospheric boundary layer is low (10–100 m) with little turbulence whereas it is high (>1,000 m) and strongly turbulent during the day. As a result, the air volume into which VPCs released from the ground are mixed is much greater by day. VPC concentrations can therefore be higher and more stable at night even though emissions are generally lower (e.g., Staudt et al., 2019). Atmospheric chemical degradation and turbulence conditions also change seasonally. During the cold season, VPC breakdown by airborne radicals and oxidants is relatively lower, the atmospheric nighttime stratification is stronger and often extends over morning hours, during which mist often settles and allows VPCs to accumulate in the boundary layer. Therefore, and considering that most VPC emissions are absent during the cold season, an olfactory signal might be more salient during winter. So far, it is unknown whether winter atmospheric conditions facilitate pheromone communication by the very few species of so-called winter moths that mate during winter. Finally, the chemical and radiative properties of the earth’s atmosphere fluctuate over longer time scales. For example, air pollution events and concentrations of associated key oxidants such as tropospheric O_3_ have been steadily increasing during the Anthropocene (Ainsworth, 2017).

## Ecological Importance of Odorscapes

Resource-indicating odor cues only represent a fraction of the odorants a searching insect will encounter. For instance, a floral odor that signals a valuable food source to pollinators is always mixed with the volatiles released by the vegetative parts of the plants. This blend is itself intertwined with volatiles emanating from the rest of the local plant community. Considerable efforts have been and still are devoted to understanding how and why insects respond to specific odor cues. On the contrary, the way insects deal with global odorscapes is rarely explicitly addressed. Background odors have often been considered as a sensory noise impairing the detection of resource-indicating cues. However, the literature contains enough examples illustrating the variety of modes by which the olfactory environment modulates insect behavior. Behavioral responses often depend on the integration of several stimuli interacting with each other either synergistically or antagonistically. The odorscape can provide a sensory context and/or convey spatial information helping insects to locate resources. The following sections will review some of these important ecological functions of the odorscapes.

### The Odorscape as a Background to the Signal: Olfactory Noise

Plant odors although varying in composition among species comprise many ubiquitous volatiles. For instance, limonene, β-ocimene, β-myrcene, and linalool are recorded in the floral scents of over 70% of plant families (Knudsen et al., 2006). Similarly, green leaf volatiles are very frequently emitted by angiosperm leaves (Hatanaka, 1993). There is therefore a high probability for any source in an ecosystem to emit odors that overlap in composition with those emitted by the plant(s) dominating the local landscape. In addition, many insect ORs respond to more than one odorant and their binding specificity decreases with increasing odorant concentration (Andersson et al., 2015). Therefore, interferences between odors *in natura* are likely, either because they share part of their constituents or because their respective volatiles activate overlapping sets of ORs. Riffell et al. (2014) have investigated the impact of a background of creosote bush odor (*Larrea tridentata*, a landscape-dominating plant) on the perception of and behavioral response to *Datura wrightii* odors (a resource-plant) by the sphingid moth *M. sexta*. They show that a background of either benzaldehyde (a compound shared by *D. wrightii* and creosote bush) or geraniol (released only by creosote bush but binding to different *M. sexta* ORs) impairs the capacity of *M. sexta* to detect and track a *D. wrightii* odor plume. Both volatiles alter the neural representation of *D. wrightii* odors by antennal lobe neurons and impair the moth’s ability to track the time structure of the stimulus. Physiologically, this impairment could result either from sensory adaptation to the background, or from an inability to discriminate signal and background odor pockets from one another (see section “The Odorscape as a Source of Spatial Information: Habitats, Trails, and Landmarks”). Background interference, if acting through sensory adaptation, would make cues or signals appear less intense, increase the minimum detectable signal concentration (Martelli et al., 2013), and therefore reduce the maximum distance from which an insect is able to track an odor plume, depending on the relative concentrations of signal and background.

Background odorants and VPCs in particular can also interfere with the chemical signals produced by insects. When tested in an arena smeared with perfume *Iridomyrmex purpureus* ants antennated both nestmate and non-nestmate individuals more frequently, compared to a control arena (Conversano et al., 2014). Insect sex pheromone signals must be less prone to background interferences than other signals because they are often composed of specific chemicals, that are detected by particularly finely tuned and highly sensitive ORs. However, in several moth species, high concentrations of some VPCs directly activate pheromone-sensitive ORNs and/or reduce their response to the sex pheromone, probably because of competition for the OR-binding site (Den Otter et al., 1978; Party et al., 2009; Hatano et al., 2015; Rouyar et al., 2015), with consequences on behavior. For instance in *Agrotis ipsilon* males, addition of a heptanal background increased the latency of flight responses to the pheromone source in a wind tunnel (Rouyar et al., 2015). In *Spodoptera littoralis* males, the sudden transition from an odor-free background to a linalool background resulted in a temporary disorientation of the insect (Party et al., 2013). In the same species, a continuous linalool background, although reducing response intensity, improved the temporal resolution of responses to pulsed pheromone stimuli by pheromone-ORNs (Rouyar et al., 2011). Coding of stimulus time structure is essential for navigation (see section “The Odorscape as a Source of Complementary Information to Insects Searching for a Mate”). Testing whether VPC backgrounds improve or impair navigation efficiency will require a detailed analysis of wind tunnel flight trajectories. Whether and under which circumstances the high VPC concentrations required in order to observe interferences with sex pheromone detection can be reached *in natura* is still an open question (Badeke et al., 2016).

### The Odorscape as a Background to the Signal: Olfactory Context

Many VOCs are emitted by a diversity of organisms in a variety of situations, and may only make sense to a given insect when encountered in a specific context. Background odors, besides their potential for interference with signal detection, may provide such a context. Indeed, many cases where the addition of contextual/background odors enhances the attractiveness or repulsiveness of a resource cue have been documented (Schröder and Hilker, 2008). A striking example of such a context dependence is that of the Scots pine, which produces a volatile bouquet that attracts egg parasitoids in response to oviposition by *Diprion pini*, a herbivorous sawfly. Compared to constitutive pine emissions, the VPC bouquet released by oviposited pine twigs only differs by significantly higher emissions of (E)-β-farnesene. However, females of the egg parasitoid *Closterocerus* (syn.: *Chrysonotomya*) *ruforum* do not respond to the sesquiterpene when presented alone, while they are attracted when (E)-β-farnesene is offered in combination with the volatile emissions of egg-free pine twigs (Mumm and Hilker, 2005). The ratio of (E)-β-farnesene to the other pine twig terpenoid volatiles (context) is key to the attraction of the parasitoid (Beyaert et al., 2010).

Recent experiments suggest that a realistic odor context may enhance the capacity of male moths to discriminate among conspecific and heterospecific mates, which is essential to maintain reproductive isolation among closely related species. When presented with calling females in a clean air background in a no-choice situation, male *S. littoralis* were attracted toward females of the sibling species *S. litura* almost as much as to conspecific females (Saveer et al., 2014). However, while the addition of host plant (cotton) odor did not affect their attraction toward conspecific signals (either synthetic full pheromone blend or calling female), it significantly reduced attraction toward heterospecific signals (either main pheromone component alone or *S. litura* calling female) (Borrero-Echeverry et al., 2018).

### The Odorscape as a Source of Complementary Information to Insects Searching for a Mate

The olfactory environment is also itself a source of information. This has been particularly studied in the context of mate selection, where odors from the surrounding plant community inform mate-searching insects on the quality of resources available around a potential mate. For instance, many publications report that volatiles emitted by host plants (i.e., plant species the insect and/or its offspring can feed on) enhance moth attraction to sex pheromones and increase their reproductive behavior (Yang et al., 2004). Looking at field trapping data, it is not always clear whether increased catches to traps lured with a combination of pheromone and host plant odor result from a true synergy or a mere addition of food-searching and mate-searching individuals. Flight attraction of *Cydia pomonella* males to blends of female sex pheromone and the host plant volatile pear ester represents a case of synergy between host plant VPCs and pheromone documented at neurophysiological and behavioral levels (Trona et al., 2013). In a wind tunnel, pear ester by itself elicited virtually no contact to source, while its addition to the sex pheromone almost doubled the proportion of moths contacting the source compared to pheromone alone. Pheromonal and host plant information are already integrated in the moth antennal lobe since the cumulus region, which receives inputs from pheromone-ORNs, was more strongly activated by the blend than by the sex pheromone alone, while pear ester alone did not activate it at all. Similarly, aggregation pheromone and plant volatiles do act synergistically on the walking locomotion of several palm tree weevil species that gather on host trees to feed and mate (Rochat et al., 2000; Said et al., 2005). In males of the polyphagous moth *S. littoralis*, mate choice is linked to host plant choice: when offered a choice between two identical sex pheromone sources placed on two different host plant species, the males went for the source located on the most preferred host plant (Thöming et al., 2013; Proffit et al., 2015).

Conversely, odors of non-host plants (i.e., low quality or unsuitable for feeding) can antagonize pheromone signals. The volatile emissions from non-host gymnosperms or toxic angiosperms reduce *S. littoralis* male attraction toward the sex pheromone (Binyameen et al., 2013) while angiosperm odors antagonize attraction of conifer-associated bark beetles toward both their aggregation pheromone and host-tree odors (Zhang et al., 1999; Zhang and Schlyter, 2004). These effects of VPCs could explain why forests with higher tree species diversity suffer lower herbivory impact (Jactel and Brockerhoff, 2007) and more generally contribute to “associational resistance,” an ecological syndrome where a good-quality host plant located near non-host or low-quality host plants is less likely to be impacted by herbivores (Jactel et al., 2011; Zakir et al., 2013). Insects can also discriminate against particularly well-defended plant individuals of their host species and modulate their response to pheromones accordingly. In *S. littoralis*, male attraction toward the sex pheromone is reduced by herbivore-induced plant volatiles, which signal high levels of anti-herbivore defenses (Hatano et al., 2015). In *Ips typographus*, attraction to the aggregation pheromone is antagonized by 1,8-cineole, a host-tree compound, whose emission rate correlates with tree resistance to *I. typographus* attacks (Andersson et al., 2010; Schiebe et al., 2012). Pheromone-ORNs and 1,8-cineole-responsive ORNs are located inside the same sensillum type in *I. typographus* antennae. A cross talk between those two ORN types was observed, such that activation of 1,8-cineole ORNs inhibits the firing of pheromone-ORNs.

### The Odorscape as a Source of Spatial Information: Habitats, Trails, and Landmarks

A resource is usually closely associated to a specific environment or habitat. Furthermore, cues emanating from the habitat are usually more salient than resource-emitted signals, such that VPCs from the habitat may allow the insect to locate broad areas within which the probability to find resources of interest is high. How insects use habitat information in their foraging behavior has long been a matter of debate. They may forage sequentially, first for habitat cues at long range, then for resource cues at shorter range. Alternatively, resource-foraging behavior may be modified or triggered in the presence of habitat cues. A detailed review of the literature pertaining to the use of habitat cues by insect can be found in Webster and Carde (2017).

**Figure 4:**
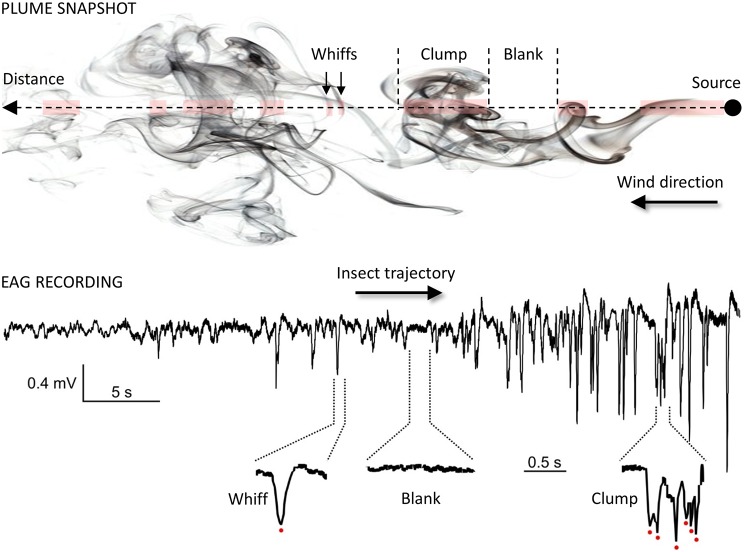
Temporal structure of the odor plume. *Plume snapshot* (upper part): the wind transports odorants away from the source along its main direction. The mean odorant concentration in the odor plume decreases with the square of the distance but local vertices mix together odorized and clean air, making the local odor concentration vary considerably around the average. As a result, odor plumes are highly intermittent signals, consisting of series of whiffs, clumps and blanks (odor/no-odor events). Foraging insects cannot rely on a chemical gradient but use the fast temporal dynamic of the plume to locate distant mates and host plants (Budick and Dickinson, 2006; Cardé and Willis, 2008). *EAG recording* (lower part): the complex structure of odor plumes can be visualized with the EAG technique (Vickers et al., 2001; Riffell et al., 2008; Nagel and Wilson, 2011) since the insect antenna responds gradually and dynamically to odor stimuli. Here, the EAG was recorded from an *Agrotis ipsilon* antenna attached to a walking red palm weevil (*Rhynchophorus ferrugineus*). The weevil was attracted to an odor source containing 10 µg of its aggregation pheromone (4-methyl-5-nonanol, ferrugineol) mixed with 10 µg of the main component of the *A. ipsilon* pheromone, (Z)7-dodecenyl acetate (Z7-12:Ac). Z7-12:Ac is thus used as a tracer of ferrugineol. The distance between the starting point of the insect and the odor source it reached was 1.75 m. Portions of the EAG recording corresponding to a whiff, a blank, and a clump of whiffs are enlarged and show single detection events (red dots).

Once within a suitable habitat, insects must navigate to locate a resource. For animals larger than a millimeter, searches take place in a turbulent environment, which adds considerable difficulties for odor source location. Indeed, as mentioned before, turbulences prevent the formation of stable odor gradients. Instead, plumes are patchy distributions of odor filaments (whiffs) interspersed with pockets of clean air (blanks; see Figure 4) (Murlis et al., 1992; Cardé and Willis, 2008; Riffell et al., 2008). Moreover, active olfactory sampling behaviors and self-generated airflows such as wing flapping, antennal flicking, and body movements modify the structure of the plume and increase the speed of encounters with odor whiffs by the antennae (Sane and Jacobson, 2006; Houot et al., 2014; Huston et al., 2015). Whiff intensity as well as whiff and blank duration are distributed according to power laws with the shortest whiffs lasting just a few milliseconds (Celani et al., 2014). While the time-averaged odor concentration decreases with the square of the distance to the source, instantaneous concentrations at a point vary rapidly over several orders of magnitude. Consequently, the time needed to obtain a reliable concentration average is much longer than the time insects actually take to make navigational decisions and the plume does not provide any directional information. Insects must respond to instantaneous odor concentration changes when locating an odor source and speed and precision of their olfactory system are crucial to accurately encode the temporal information about sensory cues. First, *Drosophila* ORNs have a very short response latency (down to 3 ms) and high precision (standard deviation below 1 ms) (Egea-Weiss et al., 2018). The latency of behavioral responses ranges from 70–85 ms after ORN response onset in *Drosophila* (Bhandawat et al., 2007; Gaudry et al., 2013) to 150–200 ms in moths (Baker and Haynes, 1987; Mafra-Neto and Cardé, 1996). Second, insects can exhibit a locomotion response to very brief odor exposures, e.g., single encounters of sex pheromone lasting 20 ms in the almond moth *Cadra cautella* (Mafra-Neto and Cardé, 1996). Third, odor space coding is linked to odor time coding. The noctuid moth *Helicoverpa zea* and the honey bee discriminate odor sources separated from each other by only a few millimeters. This remarkable capacity of spatial resolution has been postulated to rely on slight temporal differences in the arrival of odorants based on the high degree of temporal resolution of the insect olfactory system (Baker et al., 1998; Szyszka et al., 2012). Interestingly, although the recognition of odor blends requires more neuronal resources compared to single odorants, modeling studies and physiological observations indicate that multi-component odor mixtures elicit more reliable and faster olfactory coding than single odorants (Chan et al., 2018).

Navigational strategies of moths tracking a pheromone plume have been extensively investigated and there are a number of excellent reviews on the subject (e.g., Vickers, 2000, 2006; Cardé and Willis, 2008). Moths and *Drosophila* flies combine two sensory inputs to track odor plumes: the encounters of attractive odorants and the detection of the wind direction which is assessed mechanically (Bell and Wilson, 2016; Suver et al., 2019) and possibly also visually (Frye et al., 2003). For all insects tracking an odor plume, the wind direction constitutes the primary directional cue that guides them toward the source. Cockroaches, moths, and *Drosophila* appear to exploit the intermittence of odor plumes during odor-guided behavior: they surge upwind when they detect a whiff and switch to a crosswind casting behavior (moths and *Drosophila*) or to turns (cockroach) when encountering a blank (Mafra-Neto and Cardé, 1994; Willis and Avondet, 2005; Budick and Dickinson, 2006).

Contrary to their use of odor plumes as olfactory trails, we have little evidence that insects use spatial information from local odor sources as topographic-olfactory information. Evanescence of odors that can be quickly swept away by wind and changes in wind direction can make olfactory cues poorly reliable as landmarks. However, a species of desert ants, *Cataglyphis fortis*, uses olfactory cues when foraging for dead arthropods in the Tunisian salt pans (Buehlmann et al., 2015). Both the unpredictable food distribution and the high surface temperatures might account for the fact that this ant species does not use pheromone trails. *C. fortis* not only locates sparsely distributed food, or pinpoints its inconspicuous nest entrance by following odor plumes, but it also uses environmental odors as olfactory landmarks when following habitual routes.

## Plasticity and Adaptation to Complex and Variable Odorscapes

As described in the previous sections, odorscapes are both very complex and variable over time. Flexibility and integration capacities allow insects to adjust their behavior accordingly. To deal with the complexity of their olfactory environment, insects may not use all available chemical information but select the relevant cues. Besides, although many of their behaviors are innate, insects also show a remarkable plasticity through learning or physiological changes, allowing them to adjust to changes in their physiological needs or their environment. Exposure to specific VPC environments may have long-term effects on insect physiology as well as on evolutionary adaptation.

### Selective Attention and Salience Among Components of the Odorscape

With hundreds of VPCs constantly changing in concentration, odorscapes contain probably more information than the insect brain can efficiently process at any given time. Furthermore, insects cannot focus their olfaction on a specific area, like it is possible to focus vision in a direction for instance. Thus, the insect brain would be overloaded by the amount of information coming from the olfactory environment unless special adaptation such as selective attention reduces the stimulus set available to them to a subset of salient and relevant information. A growing body of literature provides behavioral and neurophysiological evidence of attention-like processes in insects for acoustic or visual stimuli (Wang et al., 2008; Nityananda, 2016). Experimental evidence for such “odor salience filters” is still scarce, probably due to the general consensus that ORs work as efficient filters performing low-level extraction of scene features according to the molecular structure of its constituents. In bees, the acquisition of a Pavlovian association between the unconditional stimulus (US), a sugar, and an odor used as conditional stimulus (CS) is delayed if the experimental bee was previously exposed to the CS without US (Fernandez et al., 2012), a process termed latent inhibition. Furthermore, not all VPCs that insects detect in the odorscape have the same importance, or salience, for them. Bumblebee performances in an associative learning protocol are linked to the salience of individual VPCs, estimated from the amplitude of electroantennogram responses (Katzenberger et al., 2013). Conditioning in *Apis mellifera* also revealed that the conditioned proboscis extension response to an odorant depends on the salience of the odorant used for conditioning, more salient (Smith, 1991) or more concentrated odorants (Wright and Smith, 2004) permitting stronger acquisitions.

### Behavioral Plasticity of Responses to Volatile Plant Compounds

Volatiles may have an intrinsic behavioral significance (valence), independently of their salience. Some signals such as pheromones have a hard-wired valence and elicit stereotypic behavioral responses. Responses to other components of the odorscape may be plastic. In polyphagous pollinators and herbivores, responses to floral or vegetative plant odors depend on learning and/or past experiences. Learning to associate plant chemical traits to the presence or absence of a reward is remarkably common and highly adaptive in generalist pollinators such as bees or moths (review in Jones and Agrawal, 2017). Indeed, the identity of the plant species providing the best nectar/pollen resource may change rapidly over the course of the flowering season, and a flower’s nectar content decreases when it gets older (Raguso and Weiss, 2015). Being able to learn new associations and to forget when a particular resource gets exhausted is therefore essential. In herbivores, ovipositing females need to lay their eggs on plant species that are suitable for their offspring’s development. While oligophagous species are able to achieve this efficiently *via* innate preferences (Gripenberg et al., 2010), polyphagous species rely more on plasticity based on their past experiences and prefer plant species they have successfully fed or mated on (Anderson and Anton, 2014; Carrasco et al., 2015).

### Long-Term Effects of Odorscapes on Insect Olfactory Behaviors

The physiological status of the receiver insect, like mating or starving, may also change the valence of an odor. *Agrotis ipsilon* immature males do not respond to the sex pheromone although they are able to detect it (Gadenne et al., 2001). The same is true for recently mated mature males. Furthermore, while the attractivity of floral odor and pheromone to unmated males adds up, presence of the sex pheromone inhibits the attraction of mated males to floral odors (Barrozo et al., 2010).

While the perception of VPCs in the odorscape can trigger fast behavioral responses, long or repeated pre-exposures to odorants may lead to long-term physiological changes through processes that may or may not involve sensory detection. In the moth *S. littoralis*, a brief pre-exposure to gustatory stimuli can change the behavioral and physiological responses to olfactory stimuli after 24 h and vice versa (Minoli et al., 2012). This long-term modulation of moth behavior correlates with modifications within the olfactory system, including up-regulation of a gene involved in olfaction (Guerrieri et al., 2012). Besides sensory effects, the physiological effects of prolonged exposures to VPCs should also be considered. Many isoprenoids have toxic effects when ingested by an insect or at high aerial concentrations. Lethality or effects on development have been well documented because of the potential use of essential oils as natural biocides, or to exploit the plant resistance to herbivory. However, non-lethal effects remain insufficiently documented. For instance, thymol, the main phenolic VPC from *Thymus vulgaris*, is used to fight the bee parasite *Varroa destructor,* but has also important effects on the bee cognitive behavior (Bergougnoux et al., 2012). The consequences of the exposure of insect populations to sub-lethal concentrations of such potentially neurotoxic volatiles would deserve further investigations.

### Evolutionary Adaptations in the Odorscape

The environments insects live in are very diverse and subject to long-term changes, including in their olfactory aspects (Figure 1). Insects have developed an olfactory system with remarkable sensitivity, specificity, and dynamics. How the evolution of this system contributes to insects’ adaptation to their lifestyle and environment is currently a hot research topic. The insect chemosensory gene families show high diversification rates, which can support fast adaptation of odorant detection capacities (Andersson et al., 2015). Antennal morphology is also very diverse and subject to selection pressures (Elgar et al., 2018). Olfactory system adaptation must be driven not only by the identity of the target signals to be detected but also by the characteristics of the olfactory environment these signals must be detected against. This remark certainly holds for OR tuning, although exploring this question will have to wait for more deorphanization data to be made available. Hansson and Stensmyr (2011) suggest that antennal morphology may reflect constraints imposed by the physical environment rather than adaptations to detect specific types of odorants. Antenna size correlates with increased sensitivity (detection surface and number of sensilla), and the sexual dimorphism in moth is a classic example where male’s larger/more elaborate antennae are an adaptation to the very low amounts of pheromone released by the females. However, attempts to correlate antennal size with pheromone volatility across species have given contradictory results (Elgar et al., 2018). Higher sensitivity may also be an advantage in environments where picking the signal is very difficult. One such example may be found in the highly specific, olfaction-driven fig tree/Agaonid pollinator mutualism. Western African populations of *Ficus sur* are pollinated by two sister species of Agaonid fig wasps (Kerdelhue et al., 1999). Although roughly sympatric, these two species differ both in their habitat preferences and their antennal morphology. *Ceratosolen silvestrianus*, mostly found in open habitats where population density of *F. sur* is high, has straight antennae. On the contrary, *C. flabellatus*, more abundant in forests where their host tree is at low density, have ramified antennae bearing more sensilla. In this case, the difficulty to pick up the signal may pertain either to the scarcity of the resource or to the physical structure of the forest habitat, which may impair the formation/persistence of navigable plumes.

Reciprocally, it is highly possible that insect community composition, especially the sensory abilities of the species composing that community as well as the identity of co-occurring plant species, influences the evolution of VPC emissions by plants and as a consequence the odorscape. For instance, the influence of pollinator- or herbivore-mediated selection on the emission of VPCs by plants has already been shown in the context of pairwise plant-species interactions (Becerra et al., 2009; Schiestl and Johnson, 2013). In addition, divergent seasonal patterns of scent emission by flowers have been revealed in a Mediterranean plant-community in relation to pollinator seasonal abundance and local plant abundance (Filella et al., 2013). More specifically, this study shows that VPC emission is higher in plant species that bloom early in the flowering period when pollinators are rare relative to flowers than in species blooming later in the season when there is a surplus of pollinators relative to flowers. The authors hypothesize that inter-specific competition for pollinator attraction might explain this variation. So far, due to the limited number of studies exploring the association between VPCs and plant-insect community structure, we have limited evidence of the effect of insect association on odorscape composition. Interestingly, a very recent study conducted at the community level pointed out an association between VPC chemical classes emitted by flowers and pollinator groups (Kantsa et al., 2019). In another study, behavioral responses of pollinator species to floral odors were found to explain a large part of the plant-pollinator network structure (Junker et al., 2010). This recent use of network-based methods to explore the importance of chemical signals in the structuring of plant-insect community will probably open the path to new discoveries on the evolution of plant-insect chemical communication.

## Odorscapes in Plant Protection and Agroecology

Manipulating the odorscapes of herbivorous pest species has already important practical implications in plant protection as an alternative to pesticides. Mating disruption methods use a synthetic sex pheromone to disturb the chemical communication between sexes. Dispensing the synthetic pheromone in the field results in the confusion of male moths which follow false trails and cannot localize females any more (Cardé and Minks, 1995). This interrupts normal mating behavior, thereby affecting chances of reproduction of pest insects. Large cultivated areas are generally treated by mating disruption to prevent introgression of mated females (Witzgall et al., 2010). Mating disruption is currently successfully used against numerous moth species in various types of crop plants, either in fields (cotton, maize), orchards (apple trees), vineyards, or even forests. Interestingly, this diversity of treated crop plants indicates that it is feasible to modify the odorscape in very different plant covers.

Modifying the odorscape implies to be able to release biologically active concentrations of odorants in the field at economically relevant costs. The success of mating disruption has promoted research for efficient dispenser technology, because the synthetic pheromone is often costly to produce. This development led, in less than 50 years, from the first hand-applied meso-dispensers to biodegradable, mechanically sprayable micro-formulations. A striking example of this development has been reviewed for the European grapevine moth, *Lobesia botrana* (Hummel, 2017). Active dispensers releasing the pheromone as puffs of aerosol at night when moths are active have been experimented, for instance against *Cydia pomonella* (McGhee et al., 2016). The decrease in moth populations obtained with these dispensers demonstrates the feasibility of a very precise control of the odorscape by adjusting the emission rates and the temporal release pattern of odorants. Still, the diffusion technology remains a bottleneck limiting the development of semiochemical uses in plant protection.

Modifying the odorscape by introducing other plant species that naturally release different VPCs can also reduce the damage caused by pest insects. Non-host plants inter-cropped with host plants decreased the oviposition of Anthomyiid flies on the hosts (Finch et al., 2003). To explain this phenomenon, it was proposed that females landed indifferently on the foliage of one or the other plant species, relying upon unspecific visual stimuli rather than on olfactory cues, but flew away from non-host plants without laying eggs because of the unsuitability of contact chemostimuli. After several errors, they finally flew away from the mixed field, this behavior resulting in a statistical reduction of the number of successful ovipositions on hosts. However, the role of non-host volatiles in oviposition deterrence has been confirmed later. For instance, methyl salicylate released by birch trees has been identified as the main factor in the reduction of mating and number of processionary moth nests on pine trees surrounded by birch trees (Jactel et al., 2011). This phenomenon is exploited in push and pull strategies, in which a pest insect is repelled from a protected crop by a repellent plant while it is attracted by plants of lesser economic value to field edges where it can be destroyed (Cook et al., 2007; Khan et al., 2010).

## Conclusions

The chemical complexity of plant volatilomes and insect olfactomes has been intensively investigated. A considerable amount of information is available regarding the identity of the volatiles mediating biotic interactions that involve insects. But we need now to grasp the complexity of the dense information networks mediated by semiochemicals. A recent analysis of a pollination network at the landscape level shows that the composition and intensity of volatile floral emissions, among other floral traits, correlate to the level of specialization of each plant species, as well as to visitation rates by the different pollinator guilds (Kantsa et al., 2018). It is striking to note that the level of complexity in their ecological role is highly variable among volatile compounds. Some semiochemicals, like most of the pheromones, are involved in specialized and confidential communication. On the other hand, single components, like β-ocimene for instance, play central roles in many biotic interactions, including pollination, and are produced and detected by diverse organisms (Farré-Armengol et al., 2017). This multifunctionality and the interweaving of olfactory interactions are serious obstacles to decipher odorscape ecological functions at multitrophic levels. It also makes it difficult to assess the impact of biotic factors (the rise of an invasive species for instance), or abiotic factors (like global warming or pollution) on olfactory communication at ecosystem scale. Using network analysis approaches in order to study how the information flows within ecosystems should overcome the apparent intricacy of odorscapes.

The ecological relevance of the concept of odorscape is stressed by the growing body of evidence indicating that the olfactory environment and other contextual information do influence the way insects respond to specific signals. Indeed, insect odorscapes are essentially multidimensional, including not only chemical identities, but also physical and temporal parameters, plus sensory, perceptual, and cognitive features. Adapting their responses to the context becomes particularly important to insects when the signal itself is ambiguous. This partly explains why insects may reliably respond to ubiquitous plant volatiles in complex olfactory scenes mixing VPCs from host and non-host plants (Meiners, 2015). Context dependence is also particularly important to consider when developing infochemicals to be applied in plant protection. For instance, genetically modified wheat constitutively producing the aphid alarm pheromone (E)-β-farnesene, although attractive to parasitoids in the lab, failed to improve aphid biocontrol in the field. This was probably because constitutive emission by the plants broke down the spatio-temporal correlation between (E)-β-farnesene and aphid presence (Bruce et al., 2015). Studying single odor signals is useful in gaining knowledge about the ecological function of these signals. But in the end, we need to consider the signals within their context in order to fully understand how infochemical networks function at the ecosystem level.

Since odorscapes are key elements of ecosystem functioning, it becomes essential to evaluate the impact of atmospheric pollution and climate change on their evolution. The study of the effect of anthropogenic volatile pollutants is just emerging and their impact on plant-to-plant and plant-to-insect communication is barely understood (Jürgens and Bischoff, 2017). There are indications that air pollution affects interactions between plants and insects beneficial to agriculture with potential consequences on plant productivity (Girling et al., 2013; Farré-Armengol et al., 2016). We need to better investigate the biological effects of atmospheric VPC reaction products on insect and plant communication (Simpraga et al., 2016). It is well established that plants modify their volatile emissions in response to biotic or abiotic stresses. Since plant metabolism responses are relatively fast compared to occurrence of visible damage, monitoring of induced VPCs could provide early alerts and allow for fast and timely implementation of remediation solutions. More studies are urgently needed, first to describe present odorscapes in a diversity of ecosystems, then to follow their evolution and evaluate how it affects the ecosystem functioning. Monitoring the odorscape composition could also serve as a reliable indicator of ecosystem quality and of biodiversity levels, a major concern in times of diminution of insect populations (Dirzo et al., 2014).

Global change is expected to have a profound impact on ecosystems, including VPC emissions and transport (Figure 1). Current knowledge on the impact of CO_2_ and temperature on plant physiology suggests a global increase in VPC emission rates as a result of climate change (Holopainen et al., 2018). How will insects respond to the resulting alterations in odorscape concentration, composition, and structure? As discussed in section “Evolutionary Adaptations in the Odorscape”, insects have developed a remarkably adaptable olfactory system, as shown by the rapid evolution of OR genes and the diversity of antennal shapes. While it is clear that OR tuning adapts to the characteristics of the signal to be detected, studies showing how the insect olfactory system adapts to specific olfactory environments, be it *via* OR tuning or antennal morphology, are needed. A combination of molecular and neuroethological methods, applied to proper models and with a sound ecological background, will allow to gain a deeper understanding of the mechanisms involved in the insect adaptation to changing environments.

Achieving this goal will require a proper description of olfactory landscapes, which depends on our capacity to isolate and identify the diverse volatile organic compounds that occur often in very small concentrations. Improvements in analytical techniques have made VPCs some of the best studied plant metabolites. Detection limits in the low ng/L range (< 1 ppbv) allow the quantification of VPCs released by single plants and are close to the detection ability of living organisms (Bicchi and Maffei, 2012). The most universal detector, the flame ionization detector is stable, linear, and offers minimum detectable amounts in the order of 0.1 ng. To bring detection limits further down, sample enrichment by dynamic head space collections on porous polymer sorbents is often used to the detriment of the temporal resolution. Yet, in natural conditions, transport of the odorant molecules by air profoundly reshapes the stimulus both spatially and temporally. The aerial concentration of VPCs undergoes considerable variation over time. It is essential to monitor this variation in order to properly describe natural odorscapes. Proton transfer reaction-mass spectrometry allows real-time trace gas monitoring at the pptv level. However, it cannot discriminate different compounds within one nominal mass, a serious limit to the apprehension of odorscape complexity. Fortunately, increasingly miniaturized set-ups combining fast trapping with fast online GC analysis and sensitivity in the ppbv range have facilitated remote field analyses. While these technical advances have created opportunities for detailed views on the time courses of VPC emissions, describing the fine temporal and spatial structure of odorscapes remains a complicated task and we still have very little insight into how it might vary across habitat types. One more argument to the necessity of studying the physics of the odorscape is the fact that notable differences between the atmospheric conditions prevailing between diurnal and nocturnal environments might have contributed to the success of olfactory communication in nocturnal insects. For instance, the lower wind, turbulence, and oxidant levels that prevail at night might facilitate the persistence of chemical trails over longer distances, and lower background VPC emissions might lead to lower olfactory noise, potentially making olfaction more reliable at night, and the cost-benefit balance for maintaining large olfactory organs more favorable (Elgar et al., 2018).

Finally, progresses in odorscape characterization will open the path to many more agronomic applications. Mating disruption, a method based on the manipulation of one critical component of moth odorscape at field scale, has offered a successful substitute to pesticides in the control of major lepidopteran pest species. At close range, repellent molecules are used to deterring hematophagous or parasite insects. Essential oil fumigations are used to eliminate pests of stored goods, but the concentrations in treated premises reach values 10^6^ times stronger than their concentrations in a natural odorscape. The huge diversity of components of essential oils provides a big reservoir of potential semiochemicals to control insects (Mossa, 2016). However, the diffusion in the field of adapted aerial concentrations of costly bioactive odorants, with different volatilities, is still a serious limitation to odorscape manipulation. New formulation technologies, which include VOCs in sprayable and biodegradable nanocapsules, will resolve many technical problems posed by field application. Besides these purely technical solutions, one might prefer natural release, for instance by plant varieties selected for their specific VPC emissions. This option will also offer the advantage of more natural solutions in agroecology.

## Author Contributions

All authors contributed to analyze the literature and write the manuscript. MR coordinated the project.

### Conflict of Interest Statement

The authors declare that the research was conducted in the absence of any commercial or financial relationships that could be construed as a potential conflict of interest.
